# Optimization of Thermoelectric Performance in *p*‐Type SnSe Crystals Through Localized Lattice Distortions and Band Convergence

**DOI:** 10.1002/advs.202411594

**Published:** 2024-12-25

**Authors:** Suniya Siddique, Ghulam Abbas, Manzar Mushaf Yaqoob, Jian Zhao, RuiHua Chen, J. Andreas Larsson, Yuede Cao, Yuexing Chen, Zhuanghao Zheng, Dongping Zhang, Fu Li

**Affiliations:** ^1^ Shenzhen Key Laboratory of Advanced Thin Films and Applications College of Physics and optoelectronic engineering Shenzhen University Shenzhen 518060 P. R. China; ^2^ Department of Physics Chemistry and Biology Linkoping University Linkoping SE‐581 83 Sweden; ^3^ Applied Physics Division of Materials Science Department of Engineering Sciences and Mathematics Luleå University of Technology Luleå 97187 Sweden; ^4^ Hefei National Laboratory for Physical Sciences at Microscale University of Science and Technology of China Hefei 230026 P. R. China; ^5^ Machano‐X Institute Applied Mechanics Laboratory Department of Engineering Mechanics Tsinghua University Beijing 100084 P. R. China

**Keywords:** band convergence, cation vacancies, lattice distortion, lattice thermal conductivity, SnSe crystals, thermoelectric material

## Abstract

Crystalline thermoelectric materials, especially SnSe crystals, have emerged as promising candidates for power generation and electronic cooling. In this study, significant enhancement in *ZT* is achieved through the combined effects of lattice distortions and band convergence in multiple electronic valence bands. Density functional theory (DFT) calculations demonstrate that cation vacancies together with Pb substitutional doping promote the band convergence and increase the density of states (DOS) near the Fermi surface of SnSe, leading to a notable increase in the Seebeck coefficient (*S*). The complex defects formed by Sn vacancies and Pb doping not only boost the Seebeck coefficient but also optimize carrier concentration (*n*_H_) and enhance electrical conductivity (*σ*), resulting in a higher power factor (*PF*). Furthermore, the localized lattice distortions induced by these defects increase phonon scattering, significantly reducing lattice thermal conductivity (*κ*_lat_) to as low as 0.29 W m^−1^ K^−1^at 773 K in Sn_0.92_Pb_0.03_Se. Consequently, these synergistic effects on phonon and electron transport contribute to a high *ZT* of 1.8. This study provides a framework for rational design of high‐performance thermoelectric materials based on first‐principles insights and experimental validation.

## Graphical abstract

Significantly enhanced figure of merit (*ZT*) is realized in *p*‐type SnSe crystals through lattice distortions and band convergence in multiple electronic valence bands. The inherently low interlayer electrical conductivity of the pristine SnSe due to its layered nature can be enhanced by introducing Sn vacancies in conjunction with Pb substitutional defects. This study provides a framework for rational design of high‐performance thermoelectric materials based on first‐principles insights and experimental validation.

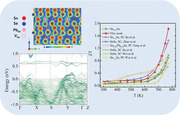

## 1 Introduction

Thermoelectric materials that enable the reversible direct solid‐state conversion of heat and electrical energy, have full potential to help tackle the energy shortage in a promising environmentally friendly manner.^[^ ^1^, ^2^, ^3^ ^]^ The conversion efficiency of the thermoelectric materials can be evaluated by the dimensionless figure of merit *ZT*, defined as *ZT* = *S* ^2^ *σT*/*κ*, where *S*, *σ*, *κ*, and *T* denote the Seebeck coefficient, electrical conductivity, total thermal conductivity, and absolute working temperature in Kelvin, respectively. The total thermal conductivity (*κ*) is the sum of the lattice thermal conductivity (*κ*_lat_) and the electronic thermal conductivity (*κ*_ele_).^[^ ^4^, ^5^ ^]^ The parameters *S*, *σ*, and *κ*_ele_ are intricately correlated with the carrier concentration (*n)* of the material. Therefore, optimizing *ZT* can be executed by strategically tuning *n* through rational doping or alloying approaches. In contrast, the correlation between *κ*_lat_ and *n* is relatively weak and can be further diminished by introducing supplementary crystal and lattice defects to scatter phonons.^[^ ^4^, ^6^ ^]^ However, these defects may concurrently scatter charge carriers, which lowers the carrier mobility and thus *σ*, making the enhancement of thermoelectric efficiency a persistent research challenge. In the past few years, the most commonly pursued strategies to improve the thermoelectric properties have included the manipulation of the density of states band engineering, such as band convergence,^[^ ^7^ ^]^ introducing the resonant impurity states near the Fermi level,^[^ ^8^ ^]^ and utilizing the energy filtering effect^[^ ^9^ ^]^ to optimize the power factor (*S* ^2^ *σ*). Additionally, enhancing the phonon scattering through all‐scale hierarchical architectures,^[^ ^10^, ^11^ ^]^ electron‐phonon transport decoupling,^[^ ^12^ ^]^ and nanostructuring^[^ ^13^, ^14^ ^]^ have been employed to reduce thermal conductivity. Recently, defect engineering has been used as a versatile and effective strategy to simultaneously optimize electrical and thermal transport properties in thermoelectric materials.^[^ ^15^, ^16^, ^17^ ^]^

In layered materials, the difference in bonding mechanisms between in‐plane and out‐of‐plane leads to unique electronic and thermal transport properties. 2D layered metal chalcogenides have recently attracted substantial attention in thermoelectrics due to their anisotropic crystal structure and low thermal conductivity.^[^ ^9^ ^]^ Among the 2D layered materials, SnSe has emerged as a promising mid‐temperature thermoelectric material due to its earth‐abundant elements, low toxicity, chemical stability, and unique transport features.^[^ ^18^ ^]^ SnSe shares a layered orthorhombic crystal structure (*Pnma*) at room‐temperature, regarded as a distorted NaCl‐type structure that supports the high Grüneisen parameters and results in the highly anharmonic and anisotropic bonding.^[^ ^19^ ^]^ The material exhibits a blend of intralayer (the strong Sn─Se covalent bonds) and interlayer weak van der Waals interactions. The presence of strong lattice anharmonicity in SnSe results in its low lattice thermal conductivity.^[^ ^18^ ^]^

Conventionally, enhancing the transport properties of ingot and sintered polycrystalline SnSe material has gained significant research interest due to its facile synthesis methods. However, in recent years, crystalline thermoelectrics have been extensively studied, primarily because of their distinct performance edges. Spectacularly, the ultralow thermal conductivity and multiple close‐to‐degenerate electronic bands enable extraordinary thermoelectric performance in both *p*‐type and *n*‐type SnSe crystals.^[^ ^19^, ^20^, ^21^, ^22^, ^23^ ^]^

In fact, polycrystalline versions of SnSe exhibit significantly lower *ZT*s values due to their higher apparent thermal conductivity compared to the reported SnSe crystals. Interestingly, recent studies have shown that polycrystalline SnSe achieves ultrahigh thermoelectric performance relative to its crystalline counterpart, attributed to the removal of the deleterious thermally conductive SnO_x_ traces from the surface of SnSe grains.^[^ ^24^, ^25^ ^]^ Single crystal samples are fundamentally devoid of grain boundaries and structural defects, thereby demonstrating higher charge carrier mobility compared to polycrystalline samples.^[^ ^23^, ^26^ ^]^ In addition to the layered structure of supporting high in‐plane carrier mobility, the unique electronic band structure enables the larger effective mass (*m* ^*^) resulting in outstanding electrical properties in the wide bandgap (≈0.86 eV) SnSe.^[^ ^27^ ^]^ Previous studies have revealed that the 3D‐charge and 2D‐phonon transport, Fermi‐surface dynamics, and manipulated layered electron‐phonon decoupling support high thermoelectric performance in *n*‐type SnSe.^[^ ^12^, ^23^, ^28^ ^]^ Whereas the manipulation of complex electronic band structure and the momentum and energy multiband synglisis enable ultrahigh *ZT* values in *p*‐type SnSe.^[^ ^27^, ^29^ ^]^ Moreover, optimizing the carrier mobility and lattice thermal conductivity by tuning the local crystal symmetry of SnSe crystals through Te alloying is found to be an effective way to enhance the *ZT* values.^[^ ^30^ ^]^ The intrinsic *p*‐type pristine SnSe is noted for its native Sn vacancies and low hole carrier concentration (≈10^17^ cm^−3^).^[^ ^30^ ^]^ In order to achieve high thermoelectric performance in SnSe, a compelling approach is to tune an appropriate carrier concentration (*n*) to optimize the electrical transport properties. In SnSe crystals, Na has been adopted as an effective dopant to raise and stabilize the carrier concentration at (3‐5) × 10^19^ cm^−3^, achieving this by lowering the Fermi level to the multiple electronic valence bands.^[^ ^27^, ^29^, ^30^, ^31^ ^]^ However, vacancy engineering is an effective way to enhance the thermoelectric performance by tuning the *n*. The intrinsic defects, such as Sn vacancies (V_Sn_), play a significant role in the excellent electronic properties of SnSe, particularly at elevated temperatures.^[^ ^32^, ^33^ ^]^ Since the formation energy of Sn vacancies is lower than that of Se vacancies, they act as the dominant defect in both *Pnma* and *Cmcm* phases. This facilitates achieving self‐doping through the induction of Sn vacancies in the SnSe matrix via off‐stoichiometric design, enabling high performance in *p*‐type SnSe.^[^ ^31^ ^]^ The Sn vacancies (as point defects) could further serve as effective phonon‐scattering sites, reducing lattice thermal conductivity by disrupting the continuity of the interlayer covalent network through both missing atoms and localized lattice disruptions.^[^ ^34^, ^35^, ^36^, ^37^ ^]^ Electronically, Pisarenko plots illustrate the inverse relationship between carrier concentration and the Seebeck coefficient.^[^ ^38^ ^]^ To mitigate the decrease in the Seebeck coefficient under carrier optimization, creating excess density of states (DOS) through band convergence is an intriguing strategy to enhance the Seebeck coefficient and, consequently, the power factor.^[^ ^39^ ^]^ Motivated by these findings, we have investigated SnSe crystals to further optimize the electrical and thermal transport properties through the combined effects of Pb substitution and the modulation of cation (V_Sn_) vacancies in the SnSe lattice.

This study presents an effective strategy for introducing cation vacancies and Pb dopants to achieve high thermoelectric performance in *p*‐type SnSe crystals, successfully synthesized by the temperature gradient method. As anticipated, the modulation of cation vacancies in the SnSe matrix effectively optimized the electrical and thermal transport properties. The further introduction of Pb doping, in conjunction with modulated Sn vacancies, facilitates valence band convergence, and restores the bandgap, thereby yielding a large Seebeck coefficient. The resultant optimization of carrier concentration concurrently enhances the electrical conductivity. Consequently, we achieve a high power factor, attributable to the combined enhancement of the electrical conductivity and the Seebeck coefficient. Moreover, dense lattice distortions, arising from these modifications, lead to an ultralow lattice thermal conductivity through strengthening phonon scattering. As a result, a competitive high *ZT* of 1.8 was attained in Sn_0.92_Pb_0.03_Se crystals at 773 K, along the in‐plane direction.

## 2 Results and Discussion

**Figure 1:**
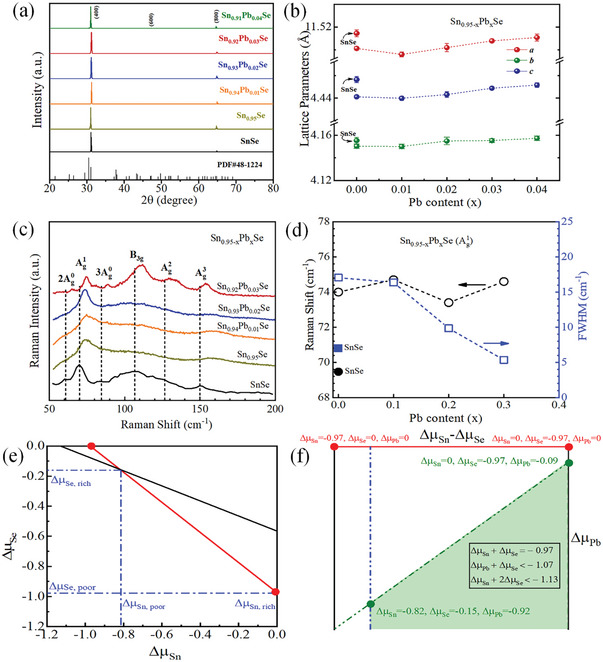
a) X‐ray diffraction (XRD) results of the cleaved surfaces from as synthesized Sn_0.95‐x_Pb_x_Se crystals, b) dependence of lattice parameters of crystals on Pb doping content (*x*), c) resonance Raman spectra of the crystals, d) Raman shift and full‐width half maxima (FWHM) variation of $A_{g}^{1}$ mode with composition variation, e) valid limit of atomic chemical potentials, where extreme Sn‐rich and Sn‐poor chemical environments represented by red, f) calculated accessible equilibrium potential region for SnSe:Pb.

**Figure** **1**a presents the X‐ray diffraction (XRD) patterns of Sn_0.95‐x_Pb_x_Se (x = 0, 0.01, 0.02, 0.03, and 0.04) and pristine SnSe. The sharp and intense (400) and (800) diffraction peaks obtained in XRD confirm that the bulk sample is a highly ordered crystal of SnSe, which indicates the crystals have unique cleavage plane along the *bc*‐plane (h00).^[^ ^36^ ^]^ All the detected diffraction peaks are well indexed to the orthorhombic crystalline structure of SnSe (JCPDS no. 48–1224) with a space group of *Pnma* orthorhombic phase. Moreover, no secondary phases were observed within the instrumental detection limit, indicating the purity and crystallinity of the synthesized crystals. Rietveld refinement of the XRD for Sn_0.95‐x_Pb_x_Se was performed to get comprehensive details of crystal structure (as illustrated in Figure S2, Supporting Information), and derived lattice parameters are shown in Figure 1b. Compared to pristine SnSe, Sn_0.95_Se manifests reduced lattice parameters, resulting in a lattice contraction. With a constant vacancy concentration of 5%, increasing the Pb content leads to an increase in the lattice parameters. This structural change can be attributed to the larger ionic radius of Pb^2+^ (1.19 Å) ions than that of Sn^2+^ (0.93 Å) ions, as well as the induced lattice distortions and strain, which will be discussed later. This also indicates that the inclusion of Pb results in a well‐doped SnSe matrix. The crystal orientation in the samples is also substantiated by the X‐ray Laue diffraction shown in Figure S1b (Supporting Information). The diffraction image clearly reveals that the crystals grow most favorably in the plane perpendicular to the *a*‐axis ([100] direction), as evidenced by the distinct Laue spots corresponding to the (100) plane that are consistent with previous studies.^[^ ^40^ ^]^ In Figure S3 (Supporting Information), the SEM image clearly exhibits the lamellar microstructure, with layers cleaved along (100) planes, supporting the XRD results of cleaved surfaces. The EDS mappings (in Figure S3, Supporting Information) indicate that no obvious element enrichment is observed and all constituent elements are homogeneously distributed in the sample, demonstrating that Pb is successfully dissolved and doped into the SnSe lattice. XPS analysis of the SnSe and Sn_0.95‐x_Pb_x_Se further confirms the effect of Pb doping on the chemical state of the samples as illustrated in Figure S4 (Supporting Information).

The resonance Raman scattering of pristine SnSe and Sn_0.95‐x_Pb_x_Se crystals has been conducted with the normalized resonance Raman spectra (RRS) presented in Figure 1c. The resonance Raman scattering experiments were carried out in the back scattering geometry, which relaxes the Raman selection rules, enabling phonons off the Brillouin zone center to contribute to the Raman scattering process. As a result, overtones and combination phonons are evident in RRS.^[^ ^41^, ^42^ ^]^ In the RRS of both pristine SnSe and Sn_0.95‐x_Pb_x_Se crystals, 2$A_{g}^{0}$, $A_{g}^{1}$, 3$A_{g}^{0}$, B_3g_, $A_{g}^{2}$, and $A_{g}^{3}$ phonon peaks are observed.^[^ ^43^ ^]^ Raman shift and an increase in full width half maxima (FWHM) of these phonon peaks are noted with the introduction of Sn vacancies and Pb substitutional dopants.

The RRS of pristine SnSe were fitted using Lorentzian‐Gaussian functions to determine the phonon peak position and its FWHM, as presented in Figure S5 (Supporting Information). The Raman shift and FWHM of the $A_{g}^{1}$ phonon peak, as obtained by fittings of RRS of pristine SnSe and Sn_0.95‐x_Pb_x_Se crystals, are plotted in Figure 1d. In Raman spectra, shift in phonon peaks correspond to the reduced mass change of the oscillating atomic pairs.^[^ ^44^ ^]^ In Sn_0.95‐x_Pb_x_Se crystals, Sn vacancies induce significant changes in the reduced mass of the constituents as compared to the Pb inclusion. Another observation is the broadening of phonon peaks with the introduction of Sn vacancies and the increase in Pb contents. When comparing the RRS of pristine SnSe with that of Sn_0.92_Pb_0.03_Se crystals, sharp phonon peaks are observed in both, which can be associated with the successful incorporation of Pb into Sn vacancy sites. The Raman line full‐width half maxima (Γ) is related to the phonon lifetime (τ) by the relation given as 1/*τ *= *Γ*/*ħ*, where *Γ* is in units of cm^−1^, *ћ* is Plank constant, which has a value of 5.3 × 10^−12^ cm^−1^ s.^[^ ^45^ ^]^ The FWHM of all the observed phonons in the RRS of Sn_0.95‐x_Pb_x_Se has decreased with increasing Pb content. The A_g_^1^ phonon peak is clearly resolved for acomposition of x = 0‐0.03, and its FWHM is plotted in Figure 1d. The FWHM has decreased significantly with increasing Pb content. The FWHM of a phonon peak is sensitive to the defects within the crystal structure.^[^ ^42^ ^]^ The decrease in the FWHM of the A_g_^1^ phonon peak indicates that the crystal structure of the Sn_0.95_Se has improved, and the number of vacancies has decreased, resembling that of pristine SnSe. The improved crystalline quality has increased the phonon lifetime of all phonons in Sn_0.95‐x_Pb_x_Se, and the estimated phonon lifetime of A_g_^1^ phonon in Sn_0.92_Pb_0.03_Se is similar to that of pristine SnSe. Our Raman results further verify the successful doping of Pb and the reduction of Sn vacancies in the Sn_0.95‐x_Pb_x_Se crystals.

As described in the methods section, the theoretically calculated value of $\Delta \;H_{SnSe}^{f}=-0.97\;eV$, which compares well with earlier theoretical (−0.96 eV)^[^ ^46^ ^]^ and experimental (−0.9 eV)^[^ ^47^ ^]^ data. Similarly, the values of $\Delta E_{\mathrm{SnSe}}^{f}=-1.3\mathrm{eV}$ and $\Delta \;H_{PbSe}^{f}=-1.07\;eV$ computed using DFT‐D3 in this work agree well with those reported in the literature.^[^ ^48^ ^]^ By simultaneously satisfying Equation (3) through 5 (as illustrated in Experimental section), we calculated the valid limits of atomic chemical potentials for Sn, Se, and Pb, which can be used in Equation (2) for examining the defect formation energies of Sn vacancies as well as the combined defect of Sn vacancy with Pb substitutional doping. For the former case, the valid limits of atomic chemical potentials are shown in Figure 1e, with extreme Sn‐rich and Sn‐poor chemical environments represented in red. Our calculated defect formation energy for extreme Sn‐poor conditions (1.24 eV/defect) clearly suggests the easy incorporation of a Sn vacancy under this Sn‐poor chemical environment as expected.^[^ ^32^ ^]^ However, in the specific case of the Sn vacancy combined with a Pb substitutional defect complex, the atomic chemical potentials satisfying Equation (5) are shown in Figure 1f, where the shaded area indicates valid limits. We have used the atomic chemical potentials shown in the shaded area under the green region. In such a chemical environment, the formation of the complex's defect for Sn vacancy combined with Pb substitutional doping at the Sn site, as realized in the experiment, shows higher formation energies under extreme Sn‐poor/Pb‐poor conditions (3.09 eV/defect) or extreme Sn‐rich/Pb‐rich (2.94 eV/defect) compared to the pristine SnSe crystal. Generally, in the case of small bandgap semiconductors, higher formation energies indicate more stringent conditions required for breaking pristine chemical bonds and forming new ones. However, such *p*‐type defects can improve the conductivity compared to the pristine system and enhance its thermoelectric properties. Furthermore, thermal activation is crucial for optimizing the carrier density. Hence, complex defects can take a synergistic role in achieving optimal carrier concentration.

**Figure 2:**
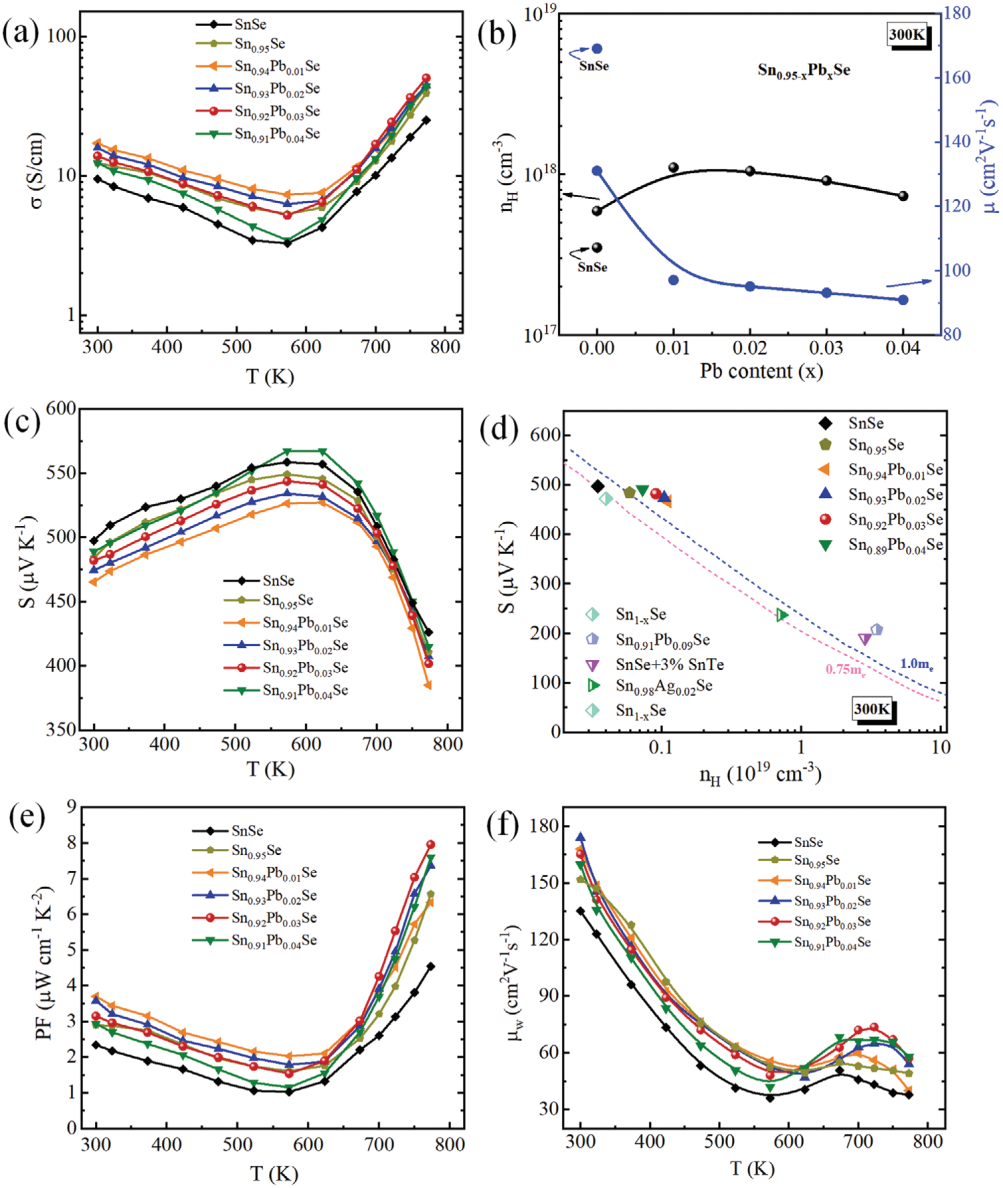
The temperature‐dependent a) electrical conductivity (*σ*), b) carrier concentration (*n*_H_) and carrier mobility (*µ*) as a function of Pb content (x), c) Seebeck coefficient (*S*), d) room‐temperature Seebeck coefficients as a function of carrier concentration for pristine SnSe and Sn_0.95‐x_Pb_x_Se, with some reported *p*‐type SnSe crystals and theoretical Pisarenko lines e) power factor (*PF*), and f) weighted mobility (*µ*_w_), for pristine SnSe and Sn_0.95‐x_Pb_x_Se crystals.

In **Figure** **2**, the electrical transport properties of pristine and Sn_0.95‐x_Pb_x_Se crystals along the in‐plane direction are depicted. As a degenerately doped semiconductor, all the samples exhibit a similar temperature‐dependent trend in electrical conductivity (*σ*), where the conduction mechanism varies with temperature as illustrated in Figure 2a. Initially, the *σ* of all the samples decreases with the increase of temperature up to 573 K, demonstrating a metallic‐like transport behavior, derived from the increased lattice vibration scattering of carriers. However, at high temperatures (beyond 573 K), the upturn of the *σ* is mainly related to the *Pnma*‐*Cmcm* phase transition. The conduction mechanism transitions to a distinctly intrinsic semiconducting behavior, which is ascribed to the thermally activated carriers taking precedence over the extrinsic carriers (from doping) in determining the conductivity of the material. Compared to the pristine SnSe crystal, the *σ* values for Sn_0.95‐x_Pb_x_Se crystals are significantly enhanced. Hall measurements were carried out to better understand the electrical transport behavior of Sn_0.92_Pb_0.03_Se crystals (as demonstrated in Figure 2b). Compared to pristine SnSe, carrier concentration (*n*_H_) is enhanced by introducing the Sn vacancies, aligning with expectations.^[^ ^33^ ^]^ Furthermore, Pb doping combined with modulated Sn vacancies shows a significant increase in *n*_H_ compared with that of SnSe, realizing 1.2 × 10^18^ cm^−3^ at room‐temperature for the Sn_0.94_Pb_0.01_Se sample. The increase in *n_H_* relative to SnSe can be attributed to the electronegativity disparity among Pb (2.33), Sn (1.96), and Se (2.55). The small electronegativity difference between Pb and Se leads to a more covalent character in their bonding than ionic, resulting in a weakened localization of electronic states that facilitates the carrier movement from the valence band to the conduction band.^[^ ^49^ ^]^ However, the *n*_H_ decreased slightly with increasing Pb content in Sn_0.95‐x_Pb_x_Se crystals. This reflects that Sn^4+^ is commonly present even in Sn^2+^ compounds, accounting for their *p*‐type conduction behavior. Conversely, Pb typically adopts the formal oxidation state of Pb^2+^ in such systems.^[^ ^50^ ^]^ Plausibly, increasing Pb doping considerably suppresses the generation of intrinsic Sn vacancies, which are the primary source of charge carriers in *p*‐type SnSe, thereby causing a lowering in *n*_H_.^[^ ^26^, ^50^ ^]^ The similar carrier concentration variation has been observed in other Pb‐doped SnSe systems.^[^ ^51^, ^52^ ^]^ The decrease in *σ* is consistent with the observed reduction in *n*_H_. Carrier mobility (*µ*) decreases with increasing Pb doping fraction, primarily due to scattering effects caused by point defects induced by Sn vacancies and Pb doping. The *n*_H_ and *µ* are tabulated in Table S1 (Supporting Information). Thus, the enhancement of *σ* in Sn_0.95‐x_Pb_x_Se crystals is attributed to the optimized hole carrier concentration despite the decrease in carrier mobility.

Figure 2c displays the temperature‐dependent Seebeck coefficient (*S*) of all the Sn_0.95‐x_Pb_x_Se crystals. The positive values of *S* confirm the *p*‐type electrical transport behavior of orthorhombic Sn_0.95‐x_Pb_x_Se crystals. As the temperature increases, the *S* shows an upward trend and reach the maximum value within the 573–623 K range, indicating the onset of bipolar conduction. Beyond this temperature (>623 K), *S* drops considerably, reflecting the increased contribution of thermally excited hole carriers. Even though the carrier concentration by modulated Sn vacancies and Pb doping in SnSe increases, the Sn_0.95‐x_Pb_x_Se crystals maintain a high *S*. Moreover, *S* increases with the increasing Pb‐doping content. The room‐temperature Seebeck coefficient (*S*) from our study and some reported doped SnSe crystals^[^ ^22^, ^29^, ^30^, ^36^ ^]^ are plotted against the carrier concentration (*n*_H_), as illustrated in Figure 2d. The Pisarenko relations (theoretical lines), estimated using a single parabolic band (SPB) model with effective masses of 0.75*m*_e_ and 1.0*m*_e_,^[^ ^53^ ^]^ are presented by pink and blue lines, respectively. The experimentally measured data of pristine SnSe from our study aligns closely with the theoretical Pisarenko line, suggesting the applicability of the SPB model in this work. Notably, the measured *S* values of Sn_0.95‐x_Pb_x_Se lie above the theoretical line, highlighting the impact of modulated Sn vacancies and Pb doping, which has substantially modified the electronic band structure of SnSe and led to an increased effective mass.

**Figure 3:**
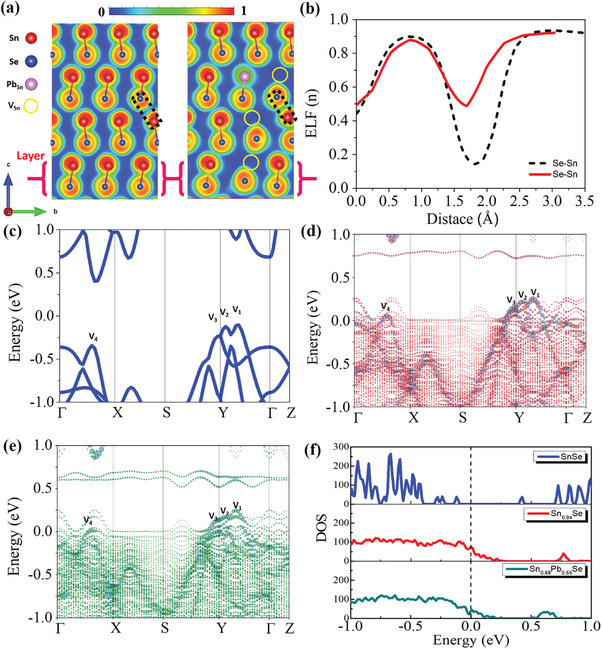
a) The cross‐sectional ELF plot of pristine SnSe and Sn_0.895_Pb_0.055_Se complex defect supercells, along with b) ELF line profiles for interlayer Se─Sn, c) the electronic band structure of pristine SnSe. The unfolded effective band structure of d) Sn_0.95_Se and e) Sn_0.895_Pb_0.055_Se along with a comparison of f) total density of states (DOS).

Ionic relaxations induced by defects result in local geometry distortions. Therefore, we commence our analysis by comparing the electron localization function (ELF) to assess the impact of lattice defects on the nature of interatomic chemical bonding.^[^ ^54^ ^]^ The ELF is a quantum mechanical measure that quantifies the likelihood of finding an electron in the proximity of another electron with parallel spin. The ELF values range from 0 to 1, where a value of 1 indicates strong electron localization, typically associated with regions of covalent bonding or the presence of lone pairs, while lower values indicate delocalization, often linked with metallic or ionic bonding. To comprehensively understand the effects of Sn vacancies along with Pb substitutional defects, DFT calculations are conducted on the low‐temperature phase (*Pnma*) of SnSe. A pre‐relaxed pristine SnSe unit cell with lattice parameters *a* = 4.14 Å, *b* = 4.61 Å, and *c* = 11.53 Å, which are in good agreement with experimental results.^[^ ^19^ ^]^ **Figure** **3**a shows cross‐sectional ELF plots for both pristine and complex defective SnSe relaxed supercells. These plots facilitate the examination of changes in interlayer and interlayer interactions. The ELF plots of pristine SnSe reveal a distinct intralayer covalent network, in contrast to the long‐range physical interactions observed between layers. The light green areas (ELF ≈ 0.5) surrounding the intralayer atomic sites indicate regions of delocalized electrons available for metal like transport. However, the ELF approaches zero, as depicted by the ELF line profile in Figure 3b, between the layers indicative of interlayer transport tunneling barriers. This also confirms that the interlayer interactions are purely physical in nature.^[^ ^55^ ^]^ Examination of the ELF plots of the supercell with defects reveals that the geometric distortions induce stronger interlayer physical interactions near Sn vacancies, as evidenced by the appearance of metallic interlayer ELF ≈ 0.5 due to strong Se═Sn interactions as detailed in the line profile of Figure 3b. This could potentially facilitate a lower barrier transport channel for electrons. In contrast, Pb substitutional sites exhibit more delocalized ELF regions compared to Sn, indicating a more metallic character, though primarily contributing to localized intralayer phase changes. Overall, the presence of such complex localized defects induces interlayer/intralayer phase changes, which are beneficial for enhanced electronic transport,^[^ ^56^ ^]^ while maintaining low thermal transport. The analysis of electronic band dispersion reveals multiple valence band minima near the Y‐Γ direction, as shown in Figure 3c. The Fermi level is set to zero as mid gap, above ≈0.3 eV of the valence band maxima. Focusing on the valence bands (the *p*‐type properties), multiple valence band maxima, labeled as V_1_ and V_2_, appear within a very narrow energy range along Y‐Γ direction in Figure 3a. In addition, a closely spaced third maximum V_3_ is observed along the S‐Y direction, and a fourth maximum, V_4_, along the Γ‐X direction. The energy differences between V_1_ and V_2_ (ΔE^V1‐V2^), V_3_ (ΔE^V1‐V4^), and V_4_ are 22, 133, and 233 meV, respectively, with an overall bandgap of 0.60 eV calculated using the PBE‐D3 semi‐local exchange correlational functional compared to 0.83 eV. Notice PBE is well known for its systematic underestimation of bandgaps, however, it still produces reasonable band dispersions. To evaluate the effects of Sn vacancies and Sn vacancies combined with Pb substitutional doping on the transport properties of SnSe, calculations using a supercell approach are required.^[^ ^57^ ^]^ Ionic relaxations induced by defects result in local geometry distortions.

In order to evaluate the impact of such localized complex defects on the electronic band structure of the SnSe, we conducted a detailed analysis of the effective band structures within a large 144‐atom supercell containing either random Sn vacancies or a complex of random Sn vacancies combined with Sn/Pb substitutional defects, as shown in Figure 3d. The effective band structure of SnSe supercells with Sn vacancies retains key features of the unit cell with multiple valence band states. The Fermi level shifts significantly into the dispersive valence band (indicative of p‐type properties), aligned with Fermi energy, as shown in Figure 3f, compared to pristine SnSe. Our 144‐atom large supercell calculations still show a significant *p*‐type character and are expected to remain so irrespective of bandgap under‐estimations.^[^ ^33^ ^]^ Notably, an increase in the bandgap to ≈ 0.70 eV is observed in the supercell containing solely Sn vacancies. In contrast, for supercells with Sn vacancies combined with Sn/Pb substitutional defect complexes, the multiple valence band states converge to within < 40 meV from the V1 state, which is positioned above the Fermi energy. In such a case, the bandgap is restored to ≈ 0.60 eV, accompanied by a minor upward shift of the Fermi level to ≈0.09 eV relative to the V1 state. This behavior is further supported by the increased Bloch‐like states created by the Sn vacancy and enhanced the density of states (DOS) near the valence band edge, as depicted in Figure 3e. Such convergence of multiple valence bands enhances the effective mass of DOS and thereby improves the Seebeck coefficient. Finally, the ELF highlights the electron density and bonding nature, which influence band alignment and convergence. Collectively, these factors impact transport properties by modifying the density of states, scattering rates, and carrier concentrations.

Overall, the inherently low interlayer electrical conductivity of the pristine SnSe due to its layered nature can be enhanced by introducing Sn vacancies, which induce geometric distortions that facilitate localized wired electron transport channels. However, the inclusion of Sn vacancies in conjunction with Pb substitutional defects helps restore the bandgap and achieve better valence band convergence, resulting in improved electrical conductivity, which is consistent with our experimental observations.

Figure 2e illustrates the temperature‐dependent power factor (*PF*) for Sn_0.95‐x_Pb_x_Se and pristine SnSe crystals. Upon introducing Sn vacancies coupled with Pb/Sn substitution, the *PF* of Sn_0.95‐x_Pb_x_Se crystals is noticeably enhanced compared with pristine SnSe. Remarkably, the sample with 3% Pb‐doped Sn_0.95_Se exhibits the highest *PF* with a value of 7.96 µW cm^−1^ K^−2^ at 773 K, which increases by 74% compared to the pure SnSe (4.54 µW cm^−1^ K^−2^) crystal. Our findings indicate that this considerable improvement in *PF* is attributed to the combined effect of optimized carrier concentration and enhanced Seebeck coefficient as a result of the modified band structure. To more intuitively assess the synergistic effect of modulated cation vacancies combined with Pb doping on the carrier transport, electronic bands, and scattering mechanism of SnSe, the temperature‐dependent weighted mobility (*µ*_w_) is calculated using the following relationship:^[^ ^6^ ^]^

(1) $$\mu_{w}=\frac{3h^{3}\sigma }{8\pi {\left(2m_{e}k_{B}T\right)}^{3/2}}\left[\frac{\exp\left[\frac{\left|S\right|}{k_{B/e}}-2\right]}{1+\exp\left[5\left(\frac{\left|S\right|}{k_{B/e}}-1\right)\right]}+\frac{\frac{3}{\pi^{2}}\frac{\left|S\right|}{k_{B/e}}}{1+\exp\left[5\frac{\left|S\right|}{k_{B/e}}-1\right]}\right]$$

where *h*, *e*, *m*_e_, and *k*_B_, represent the Planck constant, electron charge, electron mass, and Boltzmann constant, respectively. The weighted carrier mobility of Sn_0.95‐x_Pb_x_Se crystals is shown in Figure 2f. Notably, cation vacancies combined with Pb doping can effectively preserve the large weighted mobility *µ*_w_, which is attributed to the enhanced *PF* in Sn_0.95‐x_Pb_x_Se crystals.

**Figure 4:**
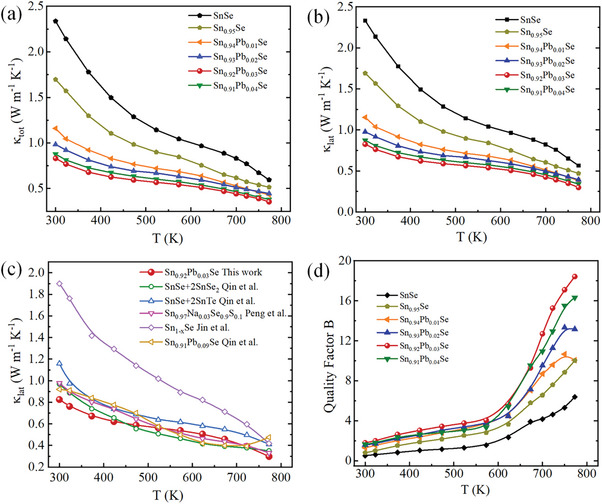
Temperature‐dependent thermal transport performance of Sn_0.95‐x_Pb_x_Se crystals a) total thermal conductivity (*κ*_tot_), b) lattice thermal conductivity (*κ*_lat_), c) comparison of *κ*_lat_ values, and d) quality factor (*B*).

**Figure** **4**a depicts the temperature dependence of the total thermal conductivity (*κ*_tot_) for all Sn_0.95‐x_Pb_x_Se crystals. *κ*_tot_ decreases with the increasing temperature primarily due to enhanced phonon‐phonon Umklapp scattering processes.^[^ ^58^ ^]^ The introduction of Pb doping combined with modulated cation vacancies in the SnSe lattice significantly reduces the thermal conductivity compared to the pure SnSe crystal. For instance, the lowest *κ*_tot_ value of 0.35 W m^−1^ K^−1^ at 773 K is realized in the Sn_0.92_Pb_0.03_Se sample. The lattice thermal conductivity (*κ*_lat_) is estimated from *κ*_lat_ = *κ*_tot_−*κ*_ele_. The electronic thermal conductivity (*κ*_ele_) is deduced by the Wiedemann‐Franz law, *κ*_ele_ = *LσT*, (Figure S6, Supporting Information). The Lorenz number (*L*) in degenerate semiconductors is considered as a function of the Fermi level, which is calculated by fitting the respective measured Seebeck coefficient and temperature within the single parabolic band model,^[^ ^59^ ^]^ as depicted in Figure S7 (Supporting Information). The variations in *κ*_ele_ are consistent with the observed trends in electrical conductivity. The results of *κ*_lat_ shown in Figure 4b indicate that the phonon contribution dominates, overshadowing the electronic part of thermal conductivity. Such low *κ*_lat_ is mainly deduced from strong bonding anharmonicity in the SnSe lattice.^[^ ^60^ ^]^ The lattice anharmonicity originates from bonding instability due to the long‐range resonant network of Se p‐bonds coupled with active Sn 5s orbitals.^[^ ^61^ ^]^ The lattice thermal conductivity of Sn_0.95‐x_Pb_x_Se crystals is complex, influenced by several factors, including phonon scattering, lattice anharmonicity, defect scattering, and phonon‐phonon interactions. The observed low values of *κ*_lat_ of Sn_0.95‐x_Pb_x_Se crystals in comparison to pristine SnSe reflect a pronounced increase in phonon scattering. At higher temperatures, intrinsic phonon‐phonon interactions prevail over the dopant‐induced effects. Crystal with composition Sn_0.92_Pb_0.03_Se has exhibited the lowest *κ*_lat_ of 0.297 W m^−1^ K^−1^ among all the synthesized samples. The defects induced by Sn vacancies combined with Pb substitution could not only strengthen the lattice anharmonicity by weakening the bonds but also increase the phonon scattering by abruptly altering the local bonding environment.^[^ ^62^ ^]^ These point defects, as the fundamental lattice imperfections, cause a strain field for phonon scattering. Notably, *κ*_lat_ of Sn_0.95‐x_Pb_x_Se crystals reduces significantly compared to previously reported values (Figure 4c).^[^ ^27^, ^29^, ^30^, ^31^, ^36^ ^]^ The reduction in lattice thermal conductivity is primarily due to the interplay of intrinsic properties and enhanced point defect scattering resulting from mass and strain field fluctuations caused by the defects.^[^ ^63^, ^64^ ^]^ By combining the temperature‐dependent *κ*_lat_ and weighted mobility *µ*_w,_ the dimensionless quality factor *B* is calculated using *B*∝(*µ*_w_/*κ*_lat_) × T^5/2^,^[^ ^65^ ^]^ to better evaluate the synergistic optimization of electrical and thermal transport in SnSe after introducing Sn vacancies with Pb doping. The significantly enhanced *B* values (as illustrated in Figure 4d) over the entire temperature range suggest that modulated cation vacancies combined with Pb doping are an effective strategy to optimize the thermoelectric performance of SnSe crystals.

**Figure 5:**
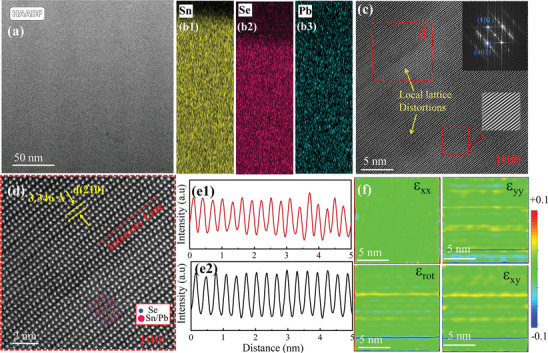
Microstructural characterization of Sn_0.92_Pb_0.03_Se crystal a) typical HAADF‐STEM image, (b1‐b3) its corresponding elemental analysis, c) magnified HAADF‐STEM image taken from (a) revealing lattice distortions in the matrix, the inset showing corresponding FFT pattern with indexed information, d) the enlarged atomic resolved HAADF‐STEM image along [110] direction taken from figure (c) marked by d, e) the line intensity profiles, (e1) taken from figure (d), and (e2) corresponds to line from HAADF‐STEM image of SnSe for comparison, f) the corresponding geometrical phase analysis (GPA) strain mapping along different directions.

To elucidate the underlying mechanism of the significantly reduced *κ*_lat_, the microstructure of the Sn_0.92_Pb_0.03_Se crystal was further characterized by scanning transmission electron microscopy (STEM). **Figure** **5**a displays the STEM image by employing a high‐angle annular dark field (HAADF) detector. HAADF image unambiguously reveals no segregated regions, confirming the absence of any secondary phase presented on the material's surface. Furthermore, an energy‐dispersive X‐ray spectroscopy (EDS) elemental mapping analysis of Figure 5a was performed to demonstrate the uniform distribution of Sn, Se, and Pb (as shown in Figures 5b1–b3). The EDS mapping confirms that Pb is uniformly dispersed in the SnSe matrix, which is reflective enough to create phonon scattering centers that hinder the thermal transport properties. Figure 5c depicts the HAADF STEM image of Sn_0.92_Pb_0.03_Se along the [110] direction. The nonuniform structural contrast indicates a lattice distortion, likely deduced from local elemental variations.^[^ ^66^ ^]^ Elemental variations disrupt the regular periodicity of the lattice, inducing strain in localized regions by introducing atoms of different size/mass into the crystal structure. Due to atomic mismatch, the incorporation of Pb dopant into the host SnSe lattice tends to induce dense lattice distortions, as illustrated in the HAADF‐STEM image in Figure 5c. These distortions are further exacerbated by the presence of distinct neighboring atoms, different bonding characteristics, and varying crystal energies.^[^ ^64^ ^]^ The corresponding fast Fourier transform (FFT) reveals the orthorhombic structure of SnSe (inset of Figure 5c). The enlarged atomic‐resolved HAADF‐STEM image along [110] direction is displayed in Figure 5d, taken from Figure 5c, indicating the typical orthorhombic *α*‐SnSe phase. Figure 5(e1) is the intensity line profile (taken along the red line in Figure 5d), illustrating the potential reasons causing the contrast difference. The line profile shows different peak intensities between different areas, reflecting the local compositional variations (such as Pb substitution and Sn vacancies). The intensity line profile corresponding to the HAADF‐STEM image of SnSe is displayed in Figure 5(e2) for comparison. Geometric phase analysis (GPA) was performed on the corresponding HAADF‐STEM image to unveil the lattice strains caused by lattice distortions. As a semi‐quantitative lattice image processing method, GPA offers valuable insights into the spatially distributed strain fields. The observed fluctuations in lattice strain suggest a wider distribution of periodic strain surrounding the lattice distortions (Figure 5f). Specifically, Figure 5f demonstrates significantly compressive strain along the vertical direction (ε_*yy*_), while the strain along the horizontal direction (ε_*xx*_) is more uniform and considerably small, indicating that strain is primarily concentrated along the y‐direction. In contrast, the strain for the pure SnSe crystal in Figure S8 (Supporting Information) along ε_*xx*_ is nearly zero, whereas the distribution along ε_*yy*_ is revealed to be uniform. Dense lattice distortions result in strong phonon scattering. The periodic strain field and lattice perturbations (distortions) induced by the size and mass disparity between Sn and Pb atoms result in substantial defect scattering, which hinders phonon transport.^[^ ^20^, ^67^ ^]^ Hence, the reduction in phonon relaxation time, primarily due to the introduction of point defects by modulated cation vacancies and Pb incorporation into the SnSe lattice, leads to significantly lowering the *κ*_lat_.

**Figure 6:**
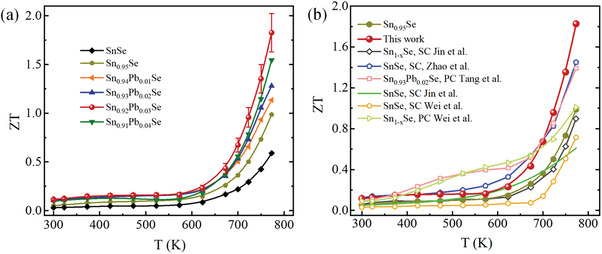
a) *ZT* as a function of temperature for Sn_0.95‐x_Pb_x_Se crystals along the *bc*‐plane direction, and b) comparison of the *ZT* with some other reported polycrystalline counterparts and crystals of the SnSe system.

**Figure** **6**a presents the calculated temperature‐dependent dimensionless figure of merit *ZT* values for the Sn_0.95‐x_Pb_x_Se crystals along the in‐plane direction. The *ZT* of Sn_0.95‐x_Pb_x_Se crystals is significantly enhanced compared to pure SnSe crystals. Mainly thanks to the simultaneous improved *PF* and reduced thermal conductivity facilitated by the partially modulated Sn vacancies and Pb doping, Sn_0.95‐x_Pb_x_Se crystals realize superior thermoelectric performances compared to pristine SnSe crystals. As presented in Figure 6a, peak *ZT* of 1.8 at 773 K is realized in the Sn_0.92_Pb_0.03_Se sample, which is ≈3 times higher than the value of pristine SnSe crystal (*ZT* ≈ 0.58 at 773 K). The peak *ZT* achieved in SnSe crystals with modulated Sn vacancies combined with Pb doping outperforms those of some other reported SnSe‐based crystals^[^ ^19^, ^68^, ^69^ ^]^ and polycrystalline counterparts^[^ ^34^, ^35^ ^]^ as presented in Figure 6b. Reproducible results of the electrical and thermal transport properties for the Sn_0.92_Pb_0.03_Se sample confirm good experimental repeatability for the high *ZT*, as illustrated in Figure S9a–d (Supporting Information).

## 3 Conclusion

In summary, we have achieved a high *ZT* of 1.8 in Sn_0.95‐x_Pb_x_Se crystals along the *bc*‐plane direction, derived from synergistically optimized electron and phonon transport. Pb doping combined with modulated cation vacancies has been found to potentially enhance the density of states, thereby yielding a large Seebeck coefficient. The electrical conductivity is improved simultaneously due to the optimization of carrier concentration. Benefiting from the enhanced electrical conductivity and large Seebeck coefficient, a high power factor of 7.88 µW cm^−1^K^−2^ at 773 K is realized for the Sn_0.92_Pb_0.03_Se crystal. The introduction of point defects due to Sn vacancies and Pb doping, along with the associated lattice distortions, leads to an ultralow lattice thermal conductivity of 0.297 W m^−1^ K^−1^. The demonstrated work provides a perspective for further improving the thermoelectric performance of SnSe crystal.

## 4 Experimental Section

In this work, the temperature gradient method was employed to synthesize Sn_0.95‐x_Pb_x_Se crystals using high‐purity raw material: Sn chunks (99.99%), Se shots (99.999%), and Pb granules (99.99%). The starting materials in appropriate nominal stoichiometric ratios for Sn_0.95‐x_Pb_x_Se (x = 0, 0.01, 0.02, 0.03, and 0.04) were loaded in conical quartz glass tubes. The tubes were evacuated and carefully flame‐sealed under the pressure of <10^−3^ Pa. The charged tube was then placed into a larger quartz tube to protect the sample from oxidation because the inner tube frequently breaks during the cooling process as the SnSe structure undergoes a phase transition with a significant thermal expansion difference between the sample and quartz tube. The double‐sealed quartz tubes were subsequently heated to 1323 K in a vertical tube furnace, allowed to be homogenized for 6 h, and then gradually cooled to 1073 K at a controlled rate of 1 K h^−1^ in order to relieve internal stress in the crystal and finally cooled down to room‐temperature. Finally, the metallic, shiny, flat‐surfaced ingots were obtained as shown in Figure S1a (Supporting Information).

The crystal structure of Sn_0.95‐x_Pb_x_Se samples was identified using an X‐ray diffraction (XRD) instrument (Bruker D8 Advance) equipped with Cu‐K_α_ radiation and a Laue diffractometer. Raman spectra were recorded in a backscattering configuration using a Renishaw Micro‐Raman Spectrometer with a laser line of 532 nm. The backscattered light was collected by a 50× objective lens, dispersed by the 1800 lines mm^−1^. Scanning Electron Microscope (SEM, Zeiss Supra55) and High Angle Annular Dark Field‐Scanning, Transmission Electron Microscopy (HAADF‐STEM, Titan 300, Waltham) equipped with an energy dispersive spectrometer (EDS) were used to assess surface morphology and composition content. The chemical state of each element in materials was determined by X‐ray photoelectron spectroscopy (XPS). In this work, the electrical and thermal transport properties of the samples were measured along the *bc*‐plane direction. The measured temperature range from 300 to 773 K is selected, as phase transition (≈800 K) may induce microcracks in the SnSe sample. The electrical conductivities (*σ*) and Seebeck coefficients (*S*) were measured simultaneously using the UlvacRiko ZEM‐3 instrument system under low pressure with an inert helium atmosphere at 300–773 K. A laser flash instrument (Netzsch LFA‐457) in an argon protection environment was used to measure thermal diffusivity (*D*). The sample's density (*ρ*) was determined with a density meter (ME204E) using the Archimedes method. The total thermal conductivity (*κ*_tot_) was calculated using the relationship *κ*_tot_ = *DC*_p_ *ρ* with specific heat (*C*_p_) values derived from the reference.^[^ ^19^ ^]^ The Hall‐carrier concentration (*n*_H_) and Hall‐mobility (*µ*) were measured by the Van der Pauw method using a Hall measurement instrument (HMS‐3000) at room‐temperature. The experimental uncertainties for the *S* and *σ* measurement are within 5%. The uncertainty of the thermal conductivity falls within ≈12%, composed of ∼5% for *D*, ≈5% for *C*_p_, and ≈2% for *ρ*. The combined uncertainty for all measurements involved in calculating the *ZT* was ≈20%.

To assess electronic structure, the first‐principles calculations are performed using the projector‐augmented wave (PAW) method as implemented in the Vienna ab‐initio Simulation Package (VASP).^[^ ^70^ ^]^ The generalized gradient approximation (CGA) of Perdew‐Bruke‐Ernzerhof (PBE)^[^ ^71^ ^]^ was adopted to describe the exchange‐correlation functional, with Grimme's semi‐empirical dispersion correction applied to properly treat the van der Waals forces. The lattice parameters and atomic coordinates were optimized until the convergence of total energy and interatomic residual force was smaller than 10^−5 ^eV and 0.01 Ev Å^−1^, respectively. A 144‐atom pristine SnSe supercell (3×3×2) was used to model systems with ≈5% Sn vacancies and a complex of 5% Sn vacancies with 5% Pb substitution to assess the impact of these defects. The Brillouin zone integration was performed using a Monkhorst‐Pack 4 × 4 × 1 k‐points mesh. Band structure analysis involved unfolding techniques post‐processed with the VASPKIT software, clarifying defect‐induced modifications to the electronic structure. To assess the combined effects of Sn vacancies and Pb substitutional doping at Sn sites, we calculated the theoretical defect formation energy of these point defects using Equation (2).^[^ ^72^ ^]^

(2) $$E^{f}=E_{d}-E_{p}+\sum_{i}n_{i}\left(E_{i}+\Delta \mu_{i}\right)$$

where *E_d_* and *E_p_* are the total energies of defective and 144‐atom pristine SnSe supercells, respectively. Δµ_*i*_ are the allowed changes in the atomic chemical potential of constituent atomic species *i*, while *E_i_* is the ground state total energy of the stable reference phase of that atomic species. *n_i_* is the number of the atoms of type *i* added to (*n_i_* < 0), or taken from (*n_i_* > 0) the host. The defect formation energy depends on the chemical potential of each element, which is related to the off‐stoichiometric degree (Se‐ or Sn‐rich). The off‐stoichiometric degree will result in different chemical potential, and thus the defect formation energy will vary accordingly. The valid upper and lower boundaries of chemical potential variation (i.e., Δµ_*Sn*_, Δµ_*Se*_, Δµ_*Pb*_ ≤ 0) can be obtained from the knowledge of the formation enthalpies of chemical systems containing the atoms Sn, Se, and Pb. For the case when SnSe is the pristine system, its formation enthalpy ($\Delta H_{SnSe}^{f}$) should satisfy

(3) $$\Delta H_{SnSe}^{f}=\Delta \mu_{Sn}+\Delta \mu_{Se}$$

On the other hand, to avoid the formation of competing phases SnSe_2_ and PbSe, the atomic chemical potentials should also confirm to the following inequalities:

(4) $$\Delta H_{SnSe_{2}}^{f}>\Delta \mu_{Sn}+2\Delta \mu_{Se}$$

(5) $$\Delta H_{PbSe}^{f}>\Delta \mu_{Pb}+\Delta \mu_{Se}$$

## Conflict of Interest

The authors declare no conflict of interest.

## Supporting information



Supporting Information

## Data Availability

The data that support the findings of this study are available from the corresponding author upon reasonable request.
