# Supplementary material for: Optimization of Thermoelectric Performance in p‐Type SnSe Crystals Through Localized Lattice Distortions and Band Convergence
Source: Adv Sci (Weinh). 2024 Dec 25;12(7):2411594. doi: 10.1002/advs.202411594 (PMC11831502; doi:10.1002/advs.202411594)
Supplement: Supplementary file 1 — Supporting Information [file ADVS-12-2411594-s001.docx]

Supporting Information

Optimization of thermoelectric performance in *p*-type SnSe crystals through localized lattice distortions and band convergence

*Suniya Siddique,^a^ Ghulam Abbas,^b,d^ Manzar Mushaf Yaqoob,^c^ Jian Zhao,^a^ RuiHua Chen,^a^ J. Andreas Larsson,^d^ Yuede Cao,^e^ Yuexing Chen,^a^ Zhuanghao Zheng,^a^ Dongping Zhang,^a^ Fu Li^a,^**

*^a^* Shenzhen Key Laboratory of Advanced Thin Films and Applications, College of Physics and Optoelectronic Engineering, Shenzhen University, Shenzhen, 518060, China.

*^b^* Department of Physics, Chemistry and Biology, Linkoping University, SE-581 83 Linkoping, Sweden.

*^c^* Hefei National Laboratory for Physical Sciences at Microscale, University of Science and Technology of China, Hefei 230026, P. R. China.

*^d^* Applied Physics, Division of Materials Science, Department of Engineering Sciences and Mathematics, Luleå University of Technology, Luleå 97187, Sweden

*^e^* Machano-X Institute, Applied Mechanics Laboratory, Department of Engineering Mechanics, Tsinghua University, Beijing 100084, China.

E-mail: lifu@szu.edu.cn

*To whom correspond should be addressed.

**Sample cutting:** As SnSe having a layered crystal structure, large enough excellent cleaved surfaces along the *bc*-plane can be obtained from grown crystalline ingots. The secured cleaved crystals were carefully cut using a diamond wire cutting machine. The cutting wire width is 0.35 mm. In order to reduce the cracking of the samples during cutting, the cutting speed was adjusted as low as 0.05 mm/min and additionally, organic oil was used. The crystalline samples were cut and finely polished into bars having dimensions of $9\times3\times3 {mm}^{3}$ for electrical transport and square-planar slices of size $9\times9\times(1.0-1.4) {mm}^{3}$ for thermal diffusivity (*D*) measurements along the *bc*-plane. Samples with dimensions $5\times5\times0.5 {mm}^{3}$were prepared to measure the Hall-coefficients (*R_H_*) _._

**X-ray back reflection Laue:** To investigate the crystal structure, perfection and crystallographic orientations, the cleavage planes of obtained Sn_0.95-x_Pb_x_Se crystals were performed on extremely valuable and accessible tool X-ray back-reflection Laue analysis. A small piece of cleaved crystal was adjusted according to the growth direction on a 3-axis goniometric head and the cleaved surface was set such facing the X-ray source. The Laue diffraction patterns were obtained through collecting diffraction lines by a photographic film on a diffractometer operating at 38.8 kV and 0.25 mA.

**Table S1.** Room temperature Hall data for pure SnSe and Sn_0.95-x_Pb_x_Se crystals.

| Sample | *n*_H_ (1x10^18^) cm^-3^ | *µ* (cm^2^ V^-1^ s^-1^) |
| --- | --- | --- |
| SnSe  Sn_0.95_Se | 0.35  0.59 | 169.078  131.04 |
| Sn_0.94_Pb_0.01_Se | 1.10 | 97.085 |
| Sn_0.93_Pb_0.02_Se | 1.05 | 95.078 |
| Sn_0.92_Pb_0.03_Se | 0.91 | 93.111 |
| Sn_0.91_Pb_0.04_Se | 0.73 | 90.901 |

**Table S2.** The calculated and measured densities of pure SnSe and Sn_0.95-x_Pb_x_Se crystals.

| Compositions | Theoretical Density  (*ρ*, *gcm*^-3^) | Actual Density  (*ρ*, *gcm*^-3^) |
| --- | --- | --- |
| SnSe | 6.165 | 5.99 |
| Sn_0.95_Se | 6.157 | 5.95 |
| Sn_0.94_Pb_0.01_Se | 6.153 | 5.90 |
| Sn_0.93_Pb_0.02_Se | 6.169 | 5.91 |
| Sn_0.92_Pb_0.03_Se | 6.171 | 5.92 |
| Sn_0.91_Pb_0.04_Se | 6.154 | 5.91 |

**Table S3**. Elemental atomic percentage determined by the EDX for SnSe and Sn_0.05-x_Pb_x_Se

| Sample | Sn (Atomic %) | Se (Atomic %) | Pb (Atomic %) |
| --- | --- | --- | --- |
| SnSe  Sn_0.94_Pb_0.01_Se | 50.140  48.068 | 49.860  51.455 | 0.00  0.476 |
| Sn_0.92_Pb_0.03_Se | 47.585 | 50.933 | 1.482 |


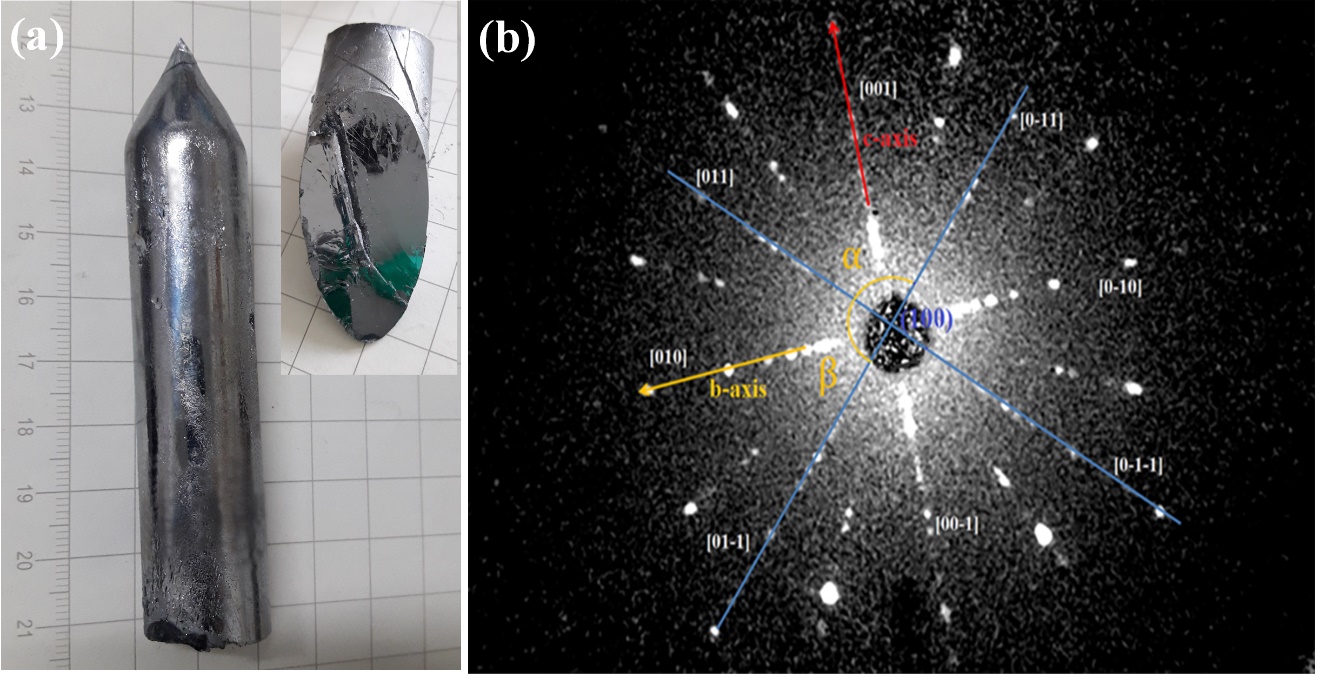


Figure S1. (a) As grown crystal with cleaved surface, (b) Laue diffraction image for the cleaved surface of Sn_0.95-x_Pb_x_Se crystals


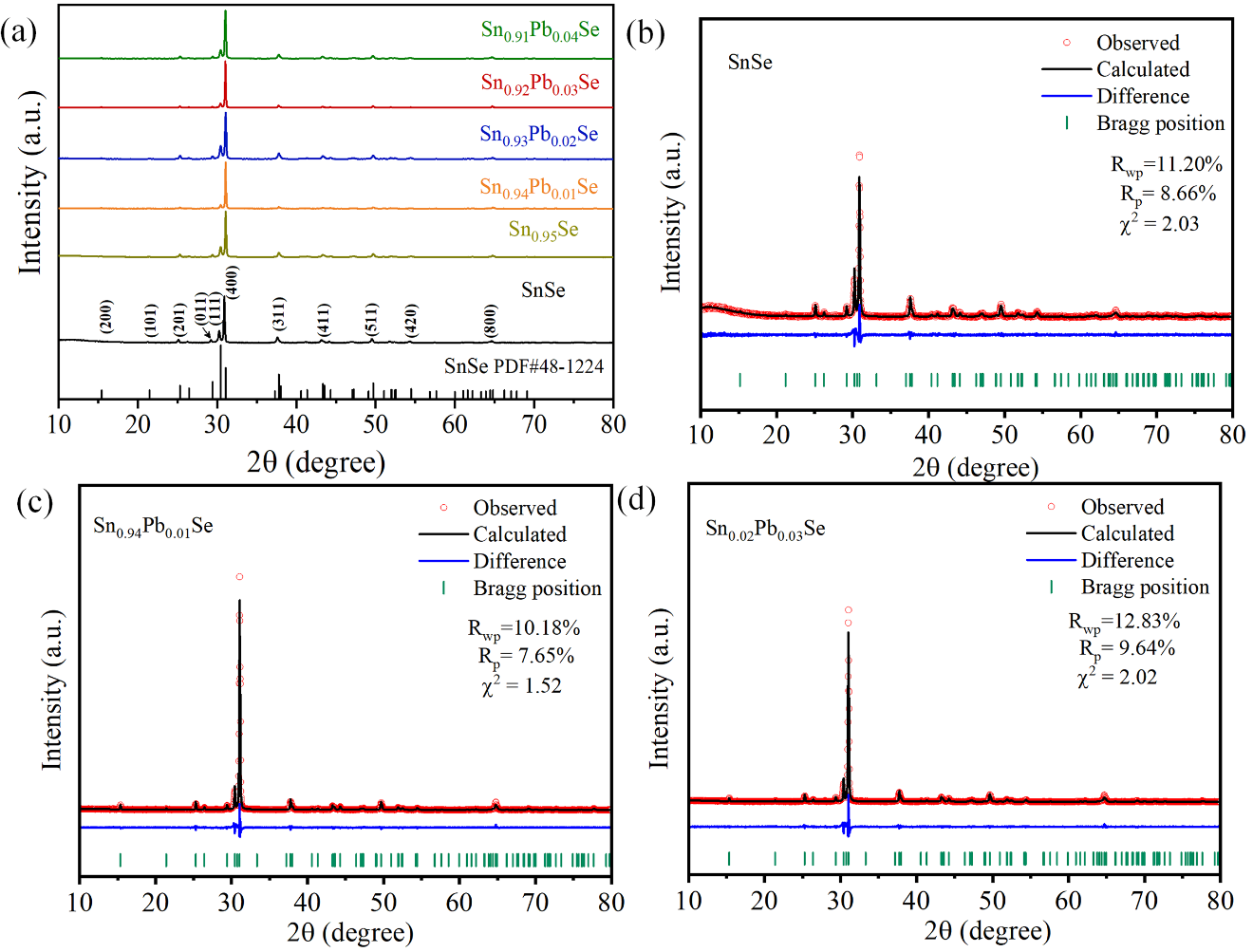


Figure S2. (a) Powder XRD of Sn_0.95-x_Pb_x_Se crystals, (b, c, d) Rietveld Refinement details for SnSe and Sn_0.92_Pb_0.03_Se.


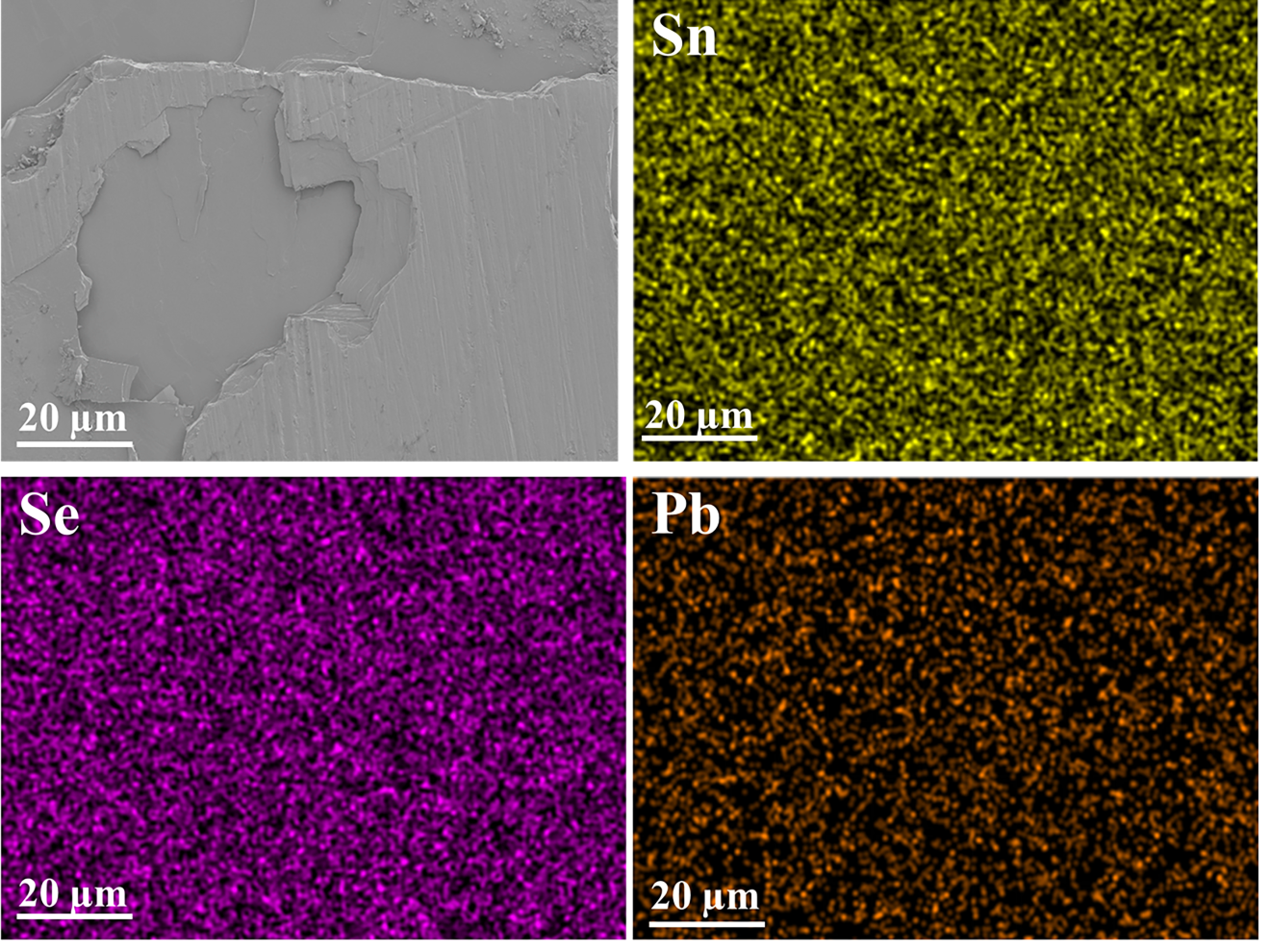


Figure S3. SEM image showing lamellar microstructure and EDS elemental mapping for Sn_0.95-x_Pb_x_Se (x=0.03) crystal.


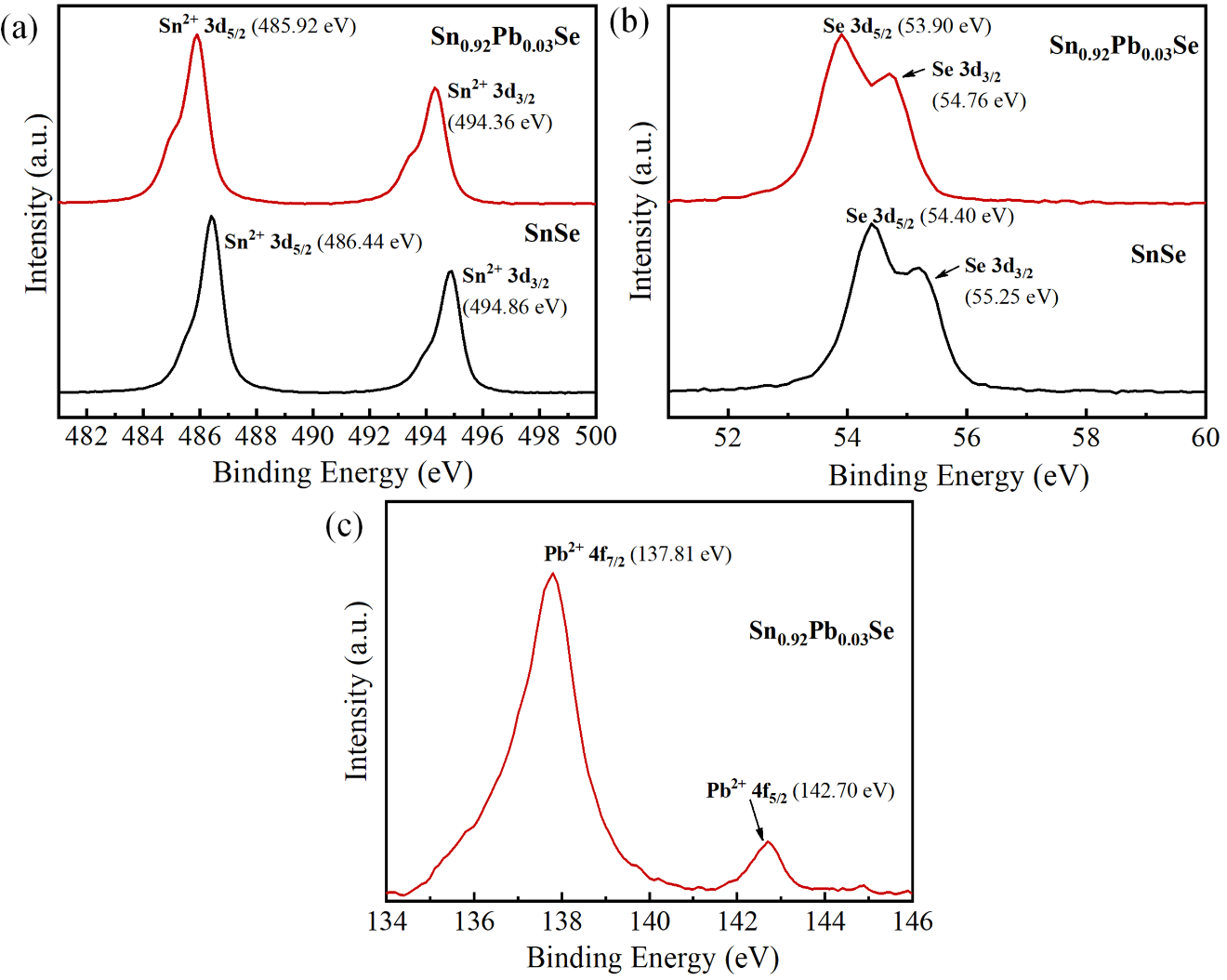


Figure S4. (a) XPS core-level spectra of (a) Sn-3d peak, (b) Se-3d peak and (c) Pb-4f from Sn_0.92_Pb_0.03_Se


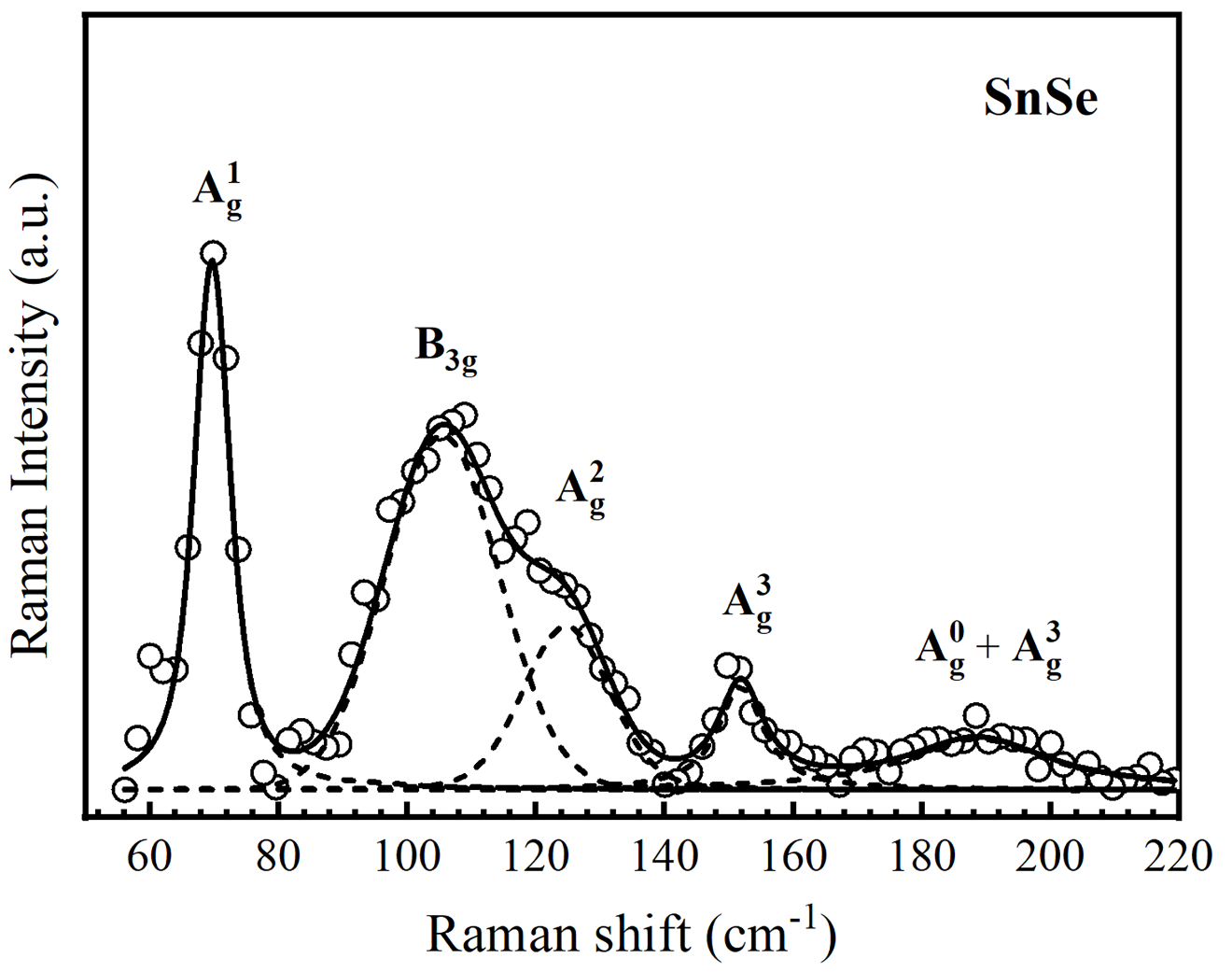


Figure S5. Lorentzian-Gaussian fitting of resonance Raman spectra of SnSe.


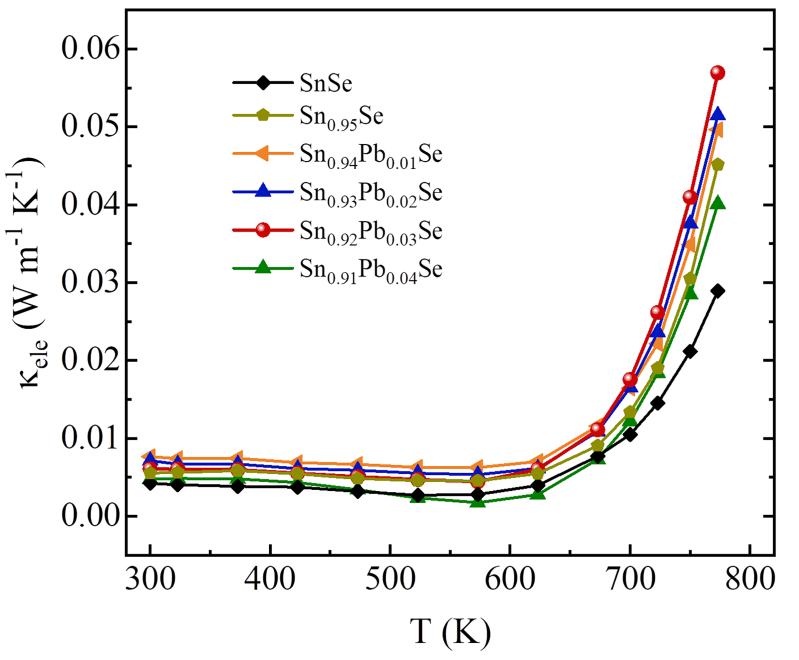


Figure S6. Electronic thermal conductivity (*κ*_ele_) of the synthesized crystals as a function of temperature.


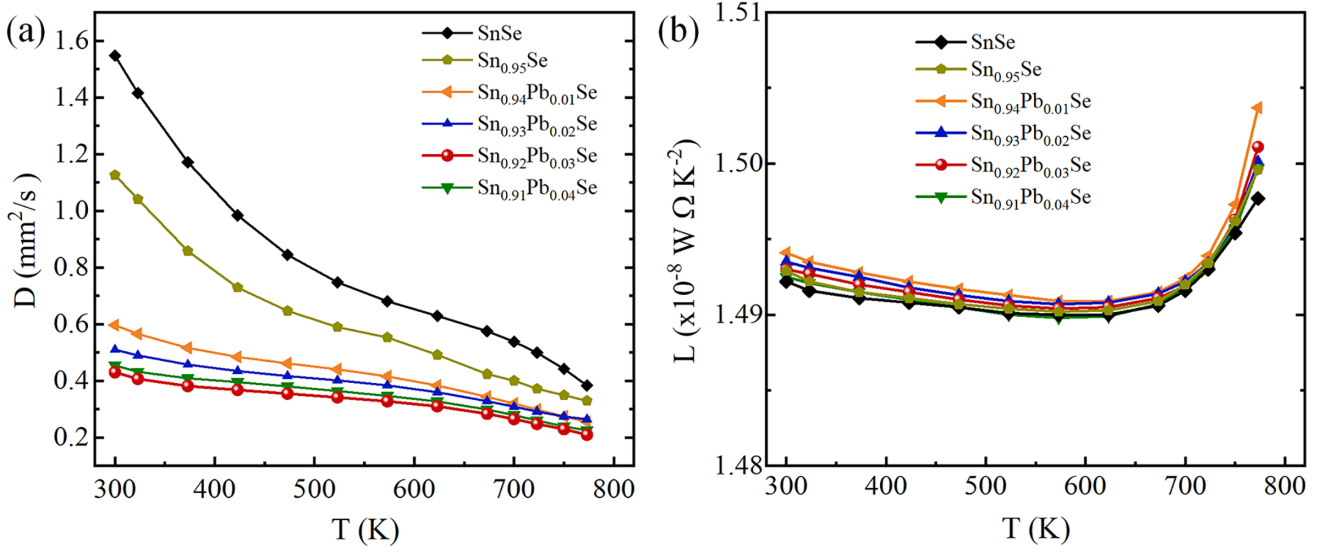


**Figure S7**. Temperature dependent (a) diffusivity (*D*), and (b) Lorenz number (*L*) of SnSe and Sn_0.95-x_Pb_x_Se crystals


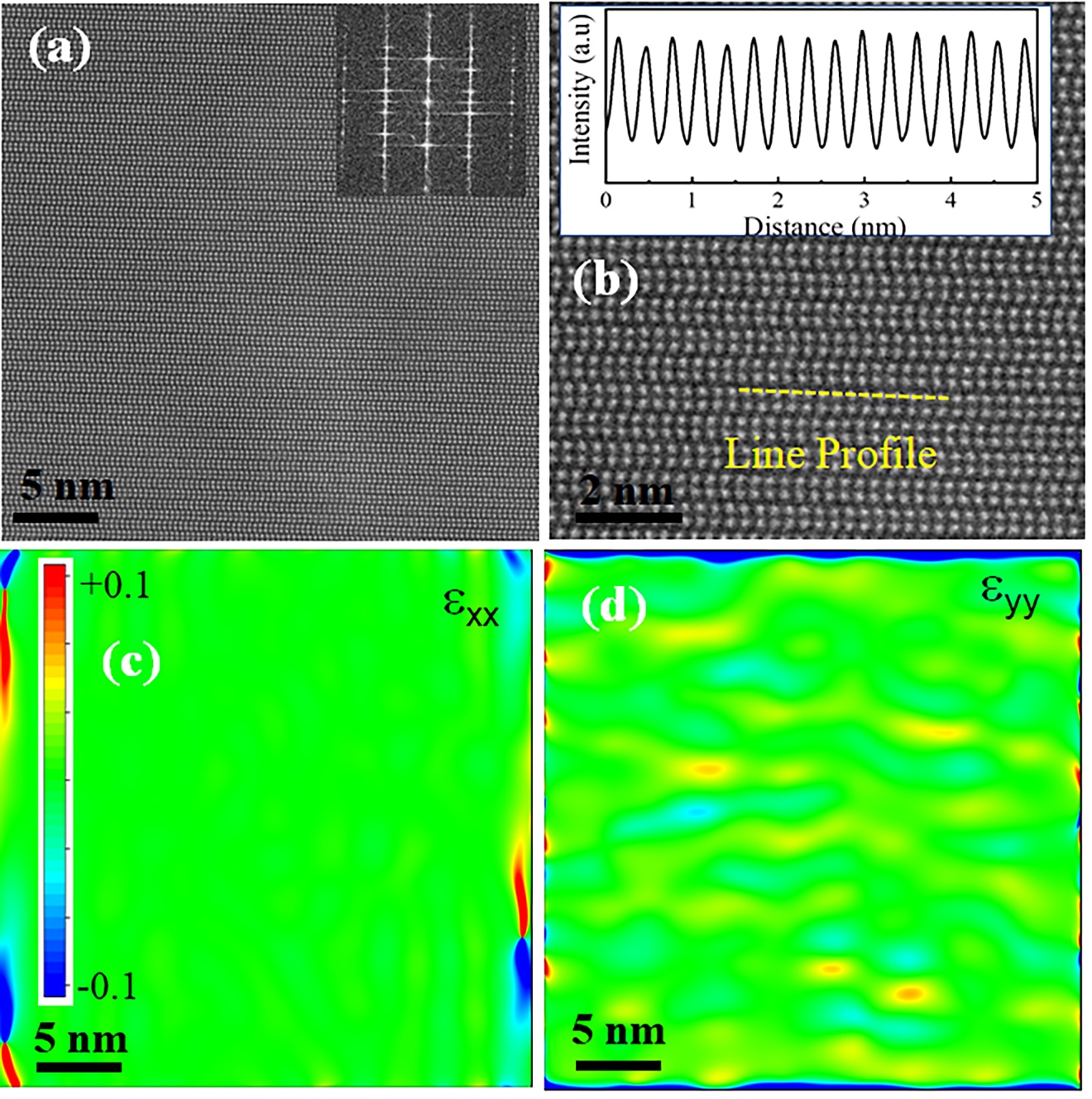


Figure S8. Microstructural characterization of SnSe crystal: (a) HAADF-STEM image with corresponding FFT in inset, (b) High-resolution STEM HAADF image taken from (a), with inset corresponding line intensity profile of yellow marked line, (c, d) the corresponding geometrical phase analysis (GPA) strain maps along the horizontal (*ε*_xx_) and vertical axis (*ε*_yy_).


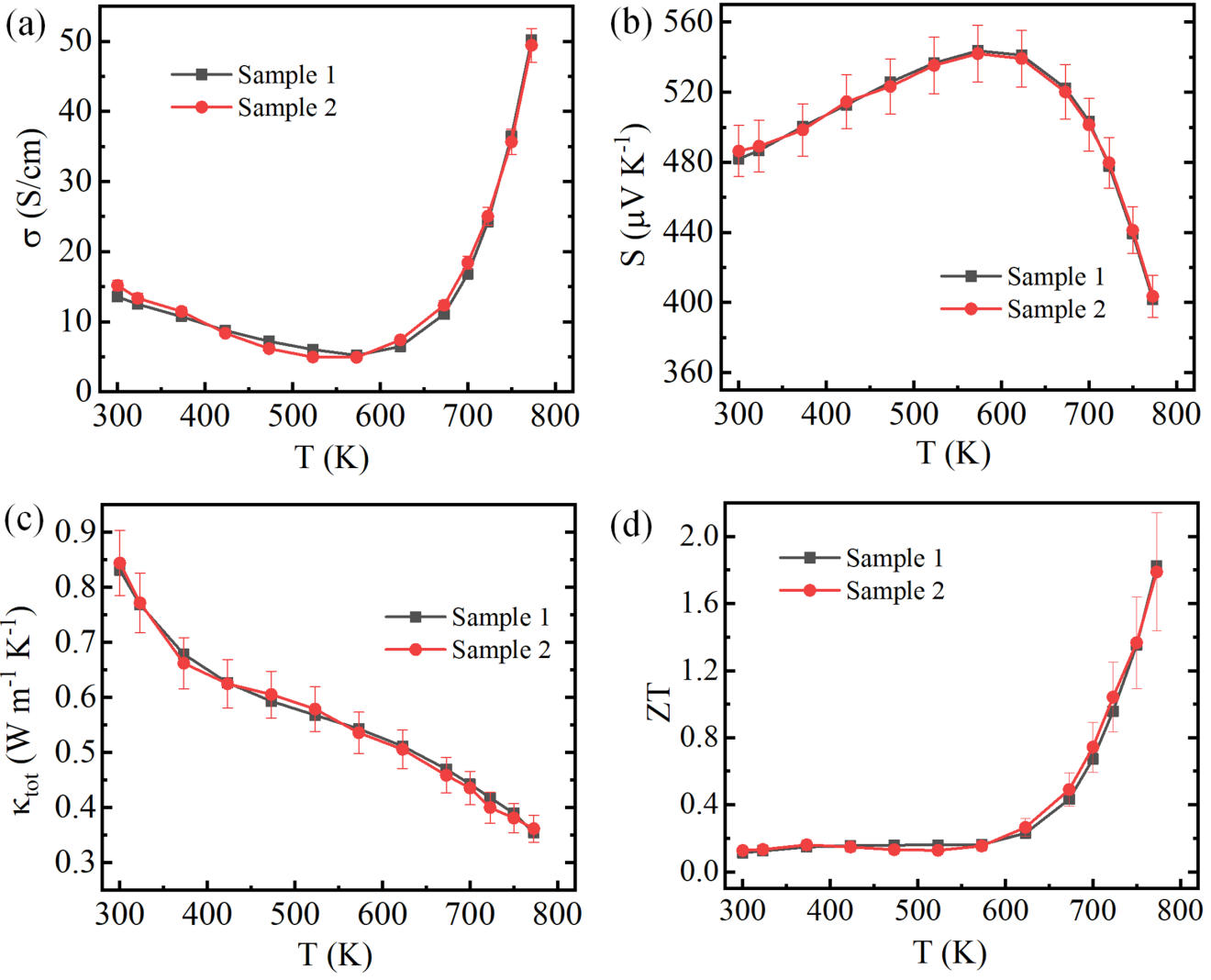


Figure S9. The reproducibility of thermoelectric properties of Sn_0.92_Pb_0.03_Se crystal, (a) electrical conductivity (*σ*), (b) Seebeck coefficient (*S*), (c) total thermal conductivity (*κ*_tot_), (d) *ZT*.
